# Minimally important difference and responsiveness to change for numerical rating scale of menstrual pain severity: a psychometric study

**DOI:** 10.3389/fpain.2025.1655464

**Published:** 2025-12-15

**Authors:** Chen X. Chen, Jingyue Wu, Chiyoung Lee, Juyoung Park, Hyochol Ahn, Lifeng Lin, Kurt Kroenke

**Affiliations:** 1 The University of Arizona College of Nursing, Tucson, AZ, United States; 2 The University of Arizona Mel and Enid Zuckerman College of Public Health, Tucson, AZ, United States; 3 Indiana University School of Medicine, Indianapolis, IN, United States; 4 Regenstrief Institute Inc, Indianapolis, IN, United States; 5 VA Center for Health Information and Communication, Indianapolis, IN, United States

**Keywords:** dysmenorrhea, pelvic pain, psychometrics, patient reported outcome measures, minimally important clinical difference

## Abstract

**Background:**

Menstrual pain affects 45%–95% of reproductive-age females and increases the risk of other chronic pain conditions. Psychometrically sound measurement tools are essential for advancing research and clinical care in menstrual pain. Numerical rating scales (NRS) are widely used to measure pain severity. However, the minimally important difference (MID) and responsiveness to change of the NRS in the context of menstrual pain are not well understood. Understanding MID and responsiveness to change helps guide the evaluation of treatment efficacy and clinical decision-making. This study evaluated the MID and responsiveness to change in the NRS, ranging from 0 to 10, for menstrual pain severity.

**Methods:**

Participants who were menstruating (aged 14–42, *N* = 100) completed two surveys 24 h apart. In both surveys, we measured menstrual pain severity (worst, least, average menstrual pain in the past 24 h, and current menstrual pain) on a 0 (no pain) to 10 (extremely severe) NRS. MIDs were estimated using distribution-based approaches (standard error of measurement and effect size) and anchor-based approaches (using symptom interference and retrospective recall of change as anchors). Responsiveness to change was evaluated using standard response means and area-under-the-curve analysis.

**Results:**

The MID estimates were close to 1 point. The NRS of menstrual pain severity was responsive to menstrual pain improvement (standard response means ranged from 0.44 to 0.61, *p* < 0.001 for between-group comparisons). Area-under-the-curve estimates ranged from 0.66 to 0.70.

**Conclusions:**

The findings can inform the design and interpretation of studies testing interventions for menstrual pain, while also guiding clinicians in monitoring and adjusting treatment.

## Introduction

Menstrual pain, or dysmenorrhea, is characterized by abdominal or pelvic pain just before or during menstruation. It affects 45%–95% of reproductive-age females and leads to impaired sleep, decreased physical activity, and absences from school and work (1, 2). Additionally, dysmenorrhea is linked to an increased risk of developing other chronic pain conditions later in life, such as irritable bowel syndrome, fibromyalgia, and non-cyclic chronic pelvic pain (3, 4). Various interventions, both pharmacological and non-pharmacological, have been proposed and tested for the treatment of dysmenorrhea (1, 5, 6). To assess the effectiveness of interventions for dysmenorrhea, it is essential to use psychometrically sound measures. Two important psychometric domains are the minimally important difference (MID) and responsiveness to change. MID is the smallest change in a health outcome that prompts a clinician to consider altering the treatment plan (7). The MID estimate helps researchers/clinicians interpret the magnitude of treatment effects, distinguish between statistically significant and clinically important changes, and serves as a benchmark for calculating statistical power (8). Responsiveness indicates how well a measure detects changes over time (8). Using measures with high responsiveness helps avoid false-negative results, reduces the need for larger sample sizes, and guides clinicians in monitoring and adjusting treatment.

Numerical rating scales (NRS) for pain severity, typically ranging from 0 to 10, are widely used in pain research, including dysmenorrhea studies (9). Research suggests that the MID for NRS ranges from 0.8 to 4 for acute pain and from 1 to 2.5 points for chronic pain (10–12), indicating changes beyond these MID thresholds are generally perceived as meaningful by patients. However, MID can be context-dependent (8). What is considered a meaningful change in one population may not apply to another (8), especially in the case of menstrual pain, which is episodic in nature. Existing studies on MID for NRS for menstrual pain severity are scarce. Two studies conducted outside the United States suggested that an approximately 3-point change represents a clinically important difference for menstrual or pelvic pain (13, 14), and a study of patients with moderate-to-severe endometriosis proposed a 4-point threshold as the clinically important difference for menstrual pain (15). However, these studies focused on clinically important differences rather than minimally important differences, which may represent smaller yet meaningful changes from the patient perspective. In addition, they either relied on highly selective clinical samples (e.g., individuals with moderate-to-severe, surgically diagnosed endometriosis) or had relatively small sample sizes (typically 200–300 participants). Given that MID estimates can be context-specific, there remains a clear need for research to establish MID values for menstrual pain.

The 0–10 NRS in measuring pain severity has been shown to be responsive and more responsive than other common tools like the visual analogue scale and verbal rating scale (16). However, the responsiveness of the NRS in the context of menstrual pain has rarely been evaluated. Pain severity can be measured using various references, including current pain, average pain, worst pain, and least pain. Each reference provides different insights into the pain experience. For instance, measuring current pain gives an immediate snapshot of the pain severity, while average pain provides an overview of the pain experienced over a period of time. Worst pain captures the highest level of pain experienced, and least pain reflects the lowest level of pain severity. Comparing how different references perform in the responsiveness measure is important for selecting the appropriate reference for research studies.

While NRS are commonly used in pain and menstrual pain studies, there has been limited research on their MID and responsiveness to change in the context of menstrual pain. This study aims to fill this gap by estimating the MID and evaluating the responsiveness to change for pain severity measured by a 0–10 NRS.

## Methods

### Design

We used a short-term longitudinal repeated measures design with a 24-hour follow-up. Participants completed a baseline survey on days 1–3 of their menstrual cycle when menstrual pain is typically most intense to reduce floor effects and maximize the chance of detecting a change (17). Menstrual pain can vary significantly across menstrual cycle days during menstruation, especially during the first few days of menstruation, making a 24-hour window clinically relevant to detect change (17, 18). A 24-hour window also minimizes recall bias, improving the reliability of MID and responsiveness estimates (9). The study was approved by the institutional review board at the Indiana University.

### Sampling

Eligible criteria were: (1) female, (2) aged 14–42, (3) able to read and converse in English, (4) currently living in the United States, and (5) having had menstrual pain in the past 6 months at enrollment, (6) menstruating and in days 1–3 of the menstrual cycle at the time of the baseline survey. The lower age limit reduced the likelihood of infrequent menstruation after menarche, while the upper age limit reduced the likelihood of enrolling perimenopausal females. The excluded age groups (≤13 and ≥43) often experience unpredictable menstrual cycles and pain patterns that may not reflect typical dysmenorrhea, potentially confounding the interpretation of psychometric results.

Participants were recruited from an opt-in survey panel managed by Qualtrics (Provo, UT). Invitations were emailed to 65,625 females aged 14–42. Participants who were on day 4 or later of their menstrual cycle were excluded to minimize floor effects, as they were less likely to be experiencing intense menstrual pain (17).

Interested individuals clicked a survey link (*n* = 1,654) and provided self-reported responses on age, sex, menstrual cycle day, and whether they had experienced menstrual pain in the past six months. Eligible individuals were shown a study information sheet. Those who decided to participate completed the survey. To ensure data quality, we embedded three attention checks (i.e., “trap questions”) in the survey and excluded participants who failed any of them. For example, one attention check stated: “*This is an attention check; please select ‘very much’ for this statement.”* We also excluded responses completed in less than one-third of the group's median completion time. To ensure adequate representation of participants in the early phase of menstruation, we implemented recruitment quotas to over-sample individuals (targeting at least one-third) who were on days 1–3 of their menstrual cycle. Out of the eligible individuals (*n* = 1,032), 836 responded to the survey, and 686 provided legitimate responses at baseline (19). Of the 260 participants surveyed on days 1–3 of their menstrual cycle at baseline, 100 provided valid follow-up responses, constituting the sample for analyses. Participant recruitment and data collection were conducted in 2019. There are no specific sample size guidelines for estimating MID and responsiveness. However, a sample size of 100 is recommended to generate precise reliability coefficients for MID estimates (20).

### Measures

The baseline survey included self-reported demographics, menstrual and gynecological information, menstrual pain severity, and dysmenorrhea symptom interference. The follow-up survey (24 h later) included menstrual pain severity, dysmenorrhea symptom interference, and a retrospective global rating of change.

#### Menstrual pain severity

The NRS was used to assess menstrual pain severity at baseline and follow-up. Its reliability and validity are supported in the literature on pain and menstrual pain (9, 13, 16). We evaluated the severity of menstrual pain by asking four questions: In the last 24 h, what number best describes your menstrual pain at its worst? At its least? On average? Right now? For each question, the scale ranged from 0 (“No pain”) to 10 (“Extremely severe”).

#### Dysmenorrhea symptom interface scale (DSI)

The DSI scale measures how dysmenorrhea symptoms interfere with daily activities. Its reliability, validity, and responsiveness are supported by previous research (19, 21, 22). We used the on-menses version of the scale at both baseline and follow-up. Participants were asked, over the last 24 h, how much their menstrual pain and menstrual gastrointestinal symptoms interfered with nine aspects of their daily lives, including physical activities, sleep, daily activities, work, concentration, enjoyment of life, leisure activities, social activities, and mood. Each item was rated on a scale of 1 (“Not at all”) to 5 (“Very much”). The DSI scale score was calculated as the average of the ratings across the nine items, with higher scores indicating greater interference (19).

#### Retrospective global rating of change

The retrospective global rating of change is widely used to assess the MID and responsiveness to change for patient-reported outcome measures (8). During the follow-up survey, participants rated how their menstrual pain had changed from the previous day on a 7-point scale with options of “Much worse”, “Somewhat worse”, “Slightly worse”, “The same”, “Slightly better”, “Somewhat better”, and “Much better”. Responses were recorded into numerical values from −3 (“Much worse”) to 3 (“Much better”).

### Data analysis

MIDs and responsiveness to change were estimated using distribution-based and anchor-based approaches, as these offer complementary insights. Distribution-based approaches rely on statistical characteristics of data, providing a more efficient measure of change by accounting for variability within the data and statistical distribution. Anchor-based approaches use an external criterion (anchor) to interpret the change score, ensuring the change in scores are meaningful and relevant to patients' experiences. There were no missing data. Data were analyzed using SAS software (Version 4.3, SAS Institute Inc., Cary, North Carolina, USA).

#### MID estimation

We estimated the MIDs using a triangulation approach that combined distribution- and anchor-based methods, as recommended in prior research (7, 23–26). Distribution-based techniques, grounded in statistical properties, offer objectivity, efficiency, and a safeguard against measurement error; however, they may overlook changes that are meaningful to patients or clinicians (26). In contrast, anchor-based methods use clinically relevant external criteria, enhancing interpretability (26). By integrating both approaches, we leveraged their complementary strengths to ensure greater robustness and clinical relevance of our findings.

##### Distribution-based approaches

We used effect size and the standard error of measurement (SEM) to derive distribution-based estimates. Effect sizes of 0.2, 0.35, and 0.5 standard deviation (SD) of baseline pain severity were calculated. According to Cohen, an effect size of 0.2 is considered small, and 0.5 is moderate (27). Research suggests that score differences greater than 0.5 standard deviations—considered a medium effect size—are likely to exceed the minimally important difference (24). Thus, we chose 0.35 SD as the MID estimates, representing a midpoint between the lower (0.2 SD) and upper (0.5 SD) boundaries of the MID estimates (24, 28).

SEM was calculated using baseline pain severity (24). SEM, defined as “the variation in the scores due to the unreliability of the scale or measure used,” was computed by standard deviation multiplied by the square root of 1 minus the reliability coefficient (29, 30). Typically, Cronbach's α is used as the reliability coefficient for a multi-item scale. However, since the NRS for pain severity is a single-item scale, we used the test-retest reliability coefficient (31). We calculated this coefficient for a subgroup of participants who reported “No change” on the retrospective global rating of change score, indicating stable menstrual pain between the baseline and the follow-up. There is no universal standard for determining how many SEM correspond to MID. One SEM has been suggested as the lower MID boundary, as a change smaller than one SEM is likely due to measurement error rather than reflecting a genuine observed change. Studies have shown that 1 SEM aligns with MID values when defined using the anchor-based approach (24, 32, 33). As a result, 1 SEM is often used as a benchmark for identifying MIDs (24, 32, 33). Two SEMs, on the other hand, have been suggested as the upper boundary of MIDs, as a 1.96 SEM or 2 SEM can represent a 95% confidence interval (CI) marginal error (24, 34, 35). Therefore, we used 1 SEM to estimate the lower bound and 2 SEM to estimate the upper bound of the MID.

##### Anchor-based approaches

Anchor-based approaches rely on an external reference (anchor) to interpret differences. These differences can be assessed cross-sectionally, by comparing clinically defined groups at a single time point, or longitudinally, by evaluating changes in scores within the same group over time (36). In this study, we used both cross-sectional and longitudinal anchor-based approaches. Anchors were selected based on their conceptual relevance to menstrual pain severity and their established use in the literature. We evaluated the correlation between an anchor and the NRS measure. A Pearson correlation of at least 0.3 indicated an appropriate anchor measure for estimating an MID (8, 24). In distinct anchor subgroups, we excluded subgroups with fewer than 10 samples from MID estimations, as estimates based on such small numbers would be unreliable (23). For each anchor-based estimate, we calculated 95% confidence intervals (CIs) using bootstrap procedures (37).

*Cross-Sectional Anchor-Based Analysis*. NRS measures were mapped onto a clinically meaningful cross-sectional anchor for between-person analysis, the DSI scale. Pearson correlations between NRS and DSI at baseline were 0.64 for worst pain, 0.53 for least pain, 0.59 for average pain, and 0.67 for current pain (*p*'s < 0.0001), supporting the use of DSI as an appropriate anchor. The MID for DSI was reported to be 0.3 point on a 1–5 scale (19). We examined the difference in NRS that corresponds to 0.3 point MID in DSI using linear regression analysis. The linearity assumption was confirmed by scatter plots.

*Longitudinal Anchor-Based Approaches*. Longitudinal anchor-based approaches address within-individual change (24). Changes in the NRS from baseline to follow-up were mapped onto DSI change score (the prospective anchor) and retrospective global rating of change score (the retrospective anchor).

For prospective anchor-based analysis, the within-person change of DSI was calculated by subtracting an individual's follow-up DSI score from their baseline DSI score. The within-person change of NRS was calculated similarly. A negative change score indicates worsened pain, while a positive change score indicates improved pain. As a 0.3 point change in DSI score has been previously demonstrated to be the MID (19), we calculated the within-person change in NRS that corresponds to 0.3 point change in DSI. Pearson correlations between the NRS change scores and DSI change scores were 0.48 for worst pain, 0.23 for least pain, 0.53 for average pain, and 0.40 for current pain. Since the correlation between the NRS change for least pain and DSI change scores was 0.23 (i.e., less than 0.3), DSI was not a proper anchor when estimating MID for the NRS for the least pain.

For retrospective anchor-based analysis*,* the global rating of changes collected at follow-up was used as the between-group anchor. This is the most widely used anchor, focusing on whether an individual has no change, improved, or worsened experience (38). Pearson correlations between NRS change scores and the retrospective global rating of changes were 0.32 for worst pain, 0.35 for least pain, 0.38 for average pain, and 0.35 for current pain (*p* < 0.0001), supporting its appropriateness as an anchor in this context. NRS change scores corresponding to one category shift (i.e., between “The same” to “Slightly better”) were used as between-group MID estimates (23, 24).

To synthesize MID estimates across different anchors, we calculated a correlation-weighted average, assigning greater weight to anchors more strongly correlated with the target measure (39).

*MID Estimation Methods Reconciliation.* As MIDs estimated by different methods can differ, the final recommended MIDs were derived from considering various distribution- and anchor-based methods (25). Distribution-based estimates were used to set approximate bounds of MID estimates. The MIDs should not be notably lower than a 0.2 effect size and one SEM to ensure they are more than a trivial difference and exceed the measurement error. At the same time, the MID estimates should not be notably higher than a 0.5 effect size or 2 SEMs to ensure that the difference is minimally important rather than moderately or substantially important (24).

#### Evaluation of responsiveness

The global rating of change was used to assess NRS's responsiveness to change. It served as an anchor to identify individuals who experienced changes from baseline to follow-up. Participants were categorized as “Improved,” “No Change,” or “Worsened” based on global rating of change. Both within-group and between-group responsiveness were evaluated.

##### Within-group responsiveness

Standardized response means (SRMs) were used to assess within-group responsiveness, reflecting the degree of change over time for each pain severity measure (e.g., worst pain, least pain, average pain, and current pain) within the “Improved,” “No Change,” and “Worsened” groups. SRM was calculated by standardizing the mean change scores using the standard deviation of these changes (40). Additionally, 95% CIs for the SRMs were generated using the bootstrapping procedure (37). According to established criteria, an absolute SRM of ≥ 0.3 indicates responsiveness (8, 41). Specifically, absolute SRMs between 0.3 and 0.5 indicate small responsiveness, values between 0.5 and 0.8 indicate moderate responsiveness, and values of 0.8 or higher reflect large responsiveness (40).

##### Between-group responsiveness

To assess between-group responsiveness, changes in scale scores across global rating of change categories were compared across “Improved”, “No change”, and “Worsened” groups. An omnibus analysis of variance (ANOVA) was conducted to compare mean changes among these three groups. *post hoc* pairwise comparisons were performed using the Tukey-Kramer test to compare (1) the “Improved” and “No Change” groups, (2) the “Worsened” and “No Change” groups, and (3) the “Improved” and “Worsened” groups, with the family-wise Type I error rate controlled at 0.05.

Receiver operating characteristic (ROC) curve analysis was also performed to quantify the ability of each measure to detect improvement. The area under the curve (AUC) represents the probability of accurately distinguishing between individuals who improved and those who did not (42, 43). For each pain severity measure, the AUC was calculated using the global rating of change as the anchor, dichotomized into “Improved” (“Slightly better,” “Somewhat better”, “Much better”) and “Not Improved” (“Much worse,” “Somewhat worse,” “Slightly worse,” or “The same”). AUC values range from 0.5 to 1.0. An AUC of greater than 0.5 would be considered meaningful, as an AUC of 0.5 represents a performance equivalent to random guessing (44).

To examine the comparative responsiveness of measures with different references (i.e., current menstrual pain, average menstrual pain, worst menstrual pain, and least menstrual pain) in detecting improvement, we statistically compared AUC values across these references.

## Results

### Study sample

Table 1 summarizes sample characteristics. Among 100 participants, the mean age was 29.3 years (SD = 6.9). Most (80%) reported using pain medication for dysmenorrhea. At baseline, the mean scores for worst, least, average in the last 24 h, and current menstrual pain were 6.21 (SD = 2.58), 3.00 (SD = 2.45), 4.94 (SD = 2.44), and 4.33 (SD = 2.92), respectively.

**Table 1:** Characteristics of participants at baseline (*N* = 100).

Baseline characteristics	Mean (SD)
Age (years)	29.28 (6.86)
Typical Length of menstrual period (days)	5.55 (1.39)
Menstrual pain severity (0–10 scale)	
Worst pain in the last 24 h	6.21 (2.58)
Least pain in the last 24 h	3.00 (2.45)
Average pain in the last 24 h	4.94 (2.44)
Current pain	4.33 (2.92)
	*n (%)*
Ethnicity	
Hispanic, Spanish, or Latino	9 (9)
Not Hispanic, Spanish, or Latino	90 (90)
Prefer not to answer	1 (1)
Race	
White	62 (62)
Black or African American	17 (17)
Asian	15 (15)
Prefer not to answer	2 (2)
Other	4 (4)
Educational Level	
8th grade or less	2 (2)
Some high school	7 (7)
High school degree or GED	13 (13)
Some college	26 (26)
2-year college degree	8 (8)
4-year college degree	30 (30)
Post-graduate degree	14 (14)
Employment Status^a^	
Student	17
Employed full-time	42
Employed part-time	10
Self-employed	7
Homemaker	14
Out of work	11
Ever Used Pain Medication Use for Menstrual Pain	80 (80)
Ever Used Hormonal Contraceptives for Menstrual Pain	41 (41)
Day of Current Menstrual Cycle	
1	19 (19)
2	32 (32)
3	49 (49)
Gynecological Conditions^a^	
Endometriosis	6 (6)
Uterine fibroids	6 (6)
Polycystic ovary syndrome	7 (7)
Pelvic inflammatory disease	4 (4)

a A participant could select more than one category.

### MID results

#### Distribution-based estimates

As shown in Table 2, an effect size of 0.35 corresponded to a change of 0.90, 0.87, 0.85, and 1.02 points, respectively, for NRS measuring worst pain, least pain, average pain, and current pain. Based on 0.2 and 0.5 effect size estimates, the MID bounds should range from 0.5 to 1.5.

**Table 2:** Minimally important difference (MID) estimates using different methods (*N* = 100).

MID estimation method	Worst pain		Least pain		Average pain		Current pain	
	Baseline	Follow up	Baseline	Follow up	Baseline	Follow up	Baseline	Follow up
Distribution-based Methods								
Effect size								
0.2 SD (Lower Bound)	0.52		0.50		0.49		0.58	
0.35 SD	0.90		0.87		0.85		1.02	
0.5 SD (Upper Bound)	1.29		1.24		1.22		1.46	
1 SEM (Lower Bound)	0.72		1.03		1.02		1.57	
2 SEM (Upper Bound)	1.44		2.06		2.04		3.14	
Anchor-based Methods								
Cross-sectional anchor:								
Dysmenorrhea Symptom Interference (95% CI)	0.46 (0.25, 0.63)		0.36 (0.15, 0.57)		0.40 (0.19, 0.57)		0.54 (0.30, 0.73)	
Longitudinal anchors (95%CI):								
Prospective: Change in Dysmenorrhea Symptom Interference	0.39 (0.16, 0.59)		0.15 (−0.04, 0.39)		0.41 (0.17, 0.71)		0.37 (0.10, 0.61)	
Retrospective: Global Rating of Change								
Slightly worse to the same^b^	0.31 (−1.58, 2.49)		0.28 (−0.92, 1.40)		−0.11 (−1.61, 1.21)		0.19 (−2.41, 2.23)	
The same to slightly better^b^	0.93 (−0.43, 2.23)		0.93 (−0.29, 2.17)		0.78 (−0.58, 2.08)		0.88 (−0.78, 2.48)	
Slightly better to much, better^a^^,^^b^	0.60 (−1.90, 2.84)		0.33 (−1.07, 1.95)		0.60 (−1.13, 3.19)		0.56 (−1.22, 2.60)	

SD, standard deviation; CI, confidence interval.

a No participant responded to “somewhat better”.

b Slightly worse: *n* = ; The same: *n* = 32; Slightly better: *n* = 30; Much better: *n * 16. The small sample sizes likely contributed to the wide confidence intervals.

One SEM ranged from 0.72 to 1.57 points (lower bound of MID) and two SEM ranged from 1.44 to 3.14 (upper bound) for the NRS measuring menstrual pain severity. Specifically, 1 SEM equated to a 0.72-point change for the worst pain, a 1.03-point change for the least pain, a 1.02-point change for average pain, and a 1.57-point change for current pain.

#### Anchor-based estimates

As seen in Table 2, cross-sectional analyses indicate that each 0.3-point difference in the baseline DSI score corresponded to a 0.46-point change in the worst pain, a 0.36-point change in the least pain, a 0.40-point change in the average pain, and a 0.54-point change in the current pain.

Based on the prospective anchor-based analysis, each 0.3-point within-person change in DSI from baseline to follow-up (i.e., MID for the DSI) corresponded to a 0.39-point within-person change in the worst pain, a 0.15-point change in the least pain, a 0.41-point change in the average pain, and a 0.37-point change in the current pain (Table 2).

Table 2 also shows the results of the retrospective anchor-based analysis using a global rating of change as the between-group change anchor. For worsened pain, the MID estimates ranged from −0.11 to 0.31 for one category of global change. For improved pain, the MID estimates ranged from 0.33 to 0.93 for one category of global change.

Triangulating across anchor-based estimates, the correlation-weighted MID was 0.51 for worst pain, 0.42 for least pain, 0.41 for average pain, and 0.51 for current pain.

#### Summary of MID estimates across approaches

**Figure 1:**
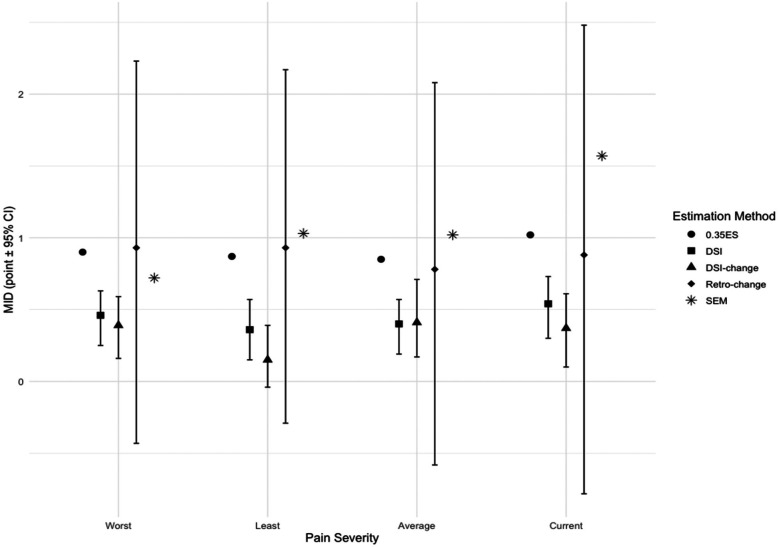
Minimally important difference estimates using different approaches. MID, Minimally Important Difference; ES, Effect Size; DSI, Dysmenorrhea Symptom Interference; Retro, Retrospective; SEM, One Standard Error of Measurement. The small subgroup for the retrospective change anchor (n ranged from 10 to 30) likely contributed to the wide confidence intervals.

Table 2 and Figure 1 illustrate the MID estimates derived from both distribution- and anchor-based approaches. Using the 1 SEM and 0.2 effect size (whichever is higher) as the approximate lower bound and 2 SEM and 0.5 effect size (whichever is lower) as the approximate higher bound, and considering anchor-based estimates, the MID for worst pain was within the range of 0.7–1.3, for least pain was in the range between 1.0 and 1.2, for average pain was within the ranged from 1.0 to 1.2, for current pain was about 1.5. In summary, the MID estimates for NRS of menstrual pain severity were close to 1 point.

### Responsiveness results

#### Within-group responsiveness

Table 3 presents the SRMs for NRS of menstrual pain severity for the improved and worsened groups. For the improved group, SRMs suggested a small to moderate and statistically significant responsiveness, ranging from 0.44 to 0.61 across NRS measures with different references. In contrast, NRS demonstrated minimal responsiveness in the worsened group, with the SRMs ranging from −0.36 to −0.24, respectively. The 95% CIs included a null value of zero, suggesting the results for the worsened group were not statistically significant.

**Table 3:** Responsiveness of within-group comparison: standardized response means.

Change of pain	Worst pain	95% CI	Least pain	95% CI	Average pain	95% CI	Current pain	95% CI
Improved	0.44^a^	(0.14, 0.77)	0.56^a^	(0.28, 0.85)	0.56^a^	(0.36, 0.84)	0.61^a^	(0.37, 0.88)
No change	−0.09	(−0.45, 0.27)	−0.08	(−0.43, 0.28)	0.12	(−0.26, 0.43)	0.09	(−0.30, 0.42)
Worsened	−0.26	(−0.65, 0.17)	−0.36	(−0.90, 0.08)	−0.25	(−0.69, 0.16)	−0.24	(−0.66, 0.18)

CI, Confidence Interval.

a Statistically significant (*p* < 0.05).

#### Between-group responsiveness

As seen in Table 4, results were statistically significant for all four NRS measures when comparing the “Improved” and “Worsened” groups. When comparing the “Improved” and “No Change” groups, results were statistically significant for the “worst pain” and “least pain” measures. However, when comparing the “No Change” and “Worsened” groups, results were not statistically significant. These findings further support the responsiveness of NRS in detecting menstrual pain improvement, but not in detecting menstrual pain worsening.

**Table 4:** Responsiveness of between group's comparison (ANOVA and tukey-kramer *post-hoc* analysis).

Menstrual symptom change comparisons	Worst pain difference between means	95% CI	Least pain difference between means	95% CI	Average pain difference between means	95% CI	Current pain difference between means	95% CI
Improved vs. No Change	1.14^a^	(0.08, 2.19)	1.04^a^	(0.17, 1.91)	0.99	(−0.02, 2.00)	1.07	(−0.13, 2.27)
Improved vs. Worsened	1.50^a^	(0.31, 2.68)	1.46^a^	(0.48, 2.44)	1.58^a^	(0.45, 2.72)	1.85^a^	(0.50, 3.20)
No Change vs. Worsened	0.36	(−0.91, 1.63)	0.42	(−0.63, 1.47)	0.60	(−0.62, 1.81)	0.78	(−0.67, 2.22)

CI, Confidence Interval.

a Statistical significant (*p* < 0.05).

**Figure 2:**
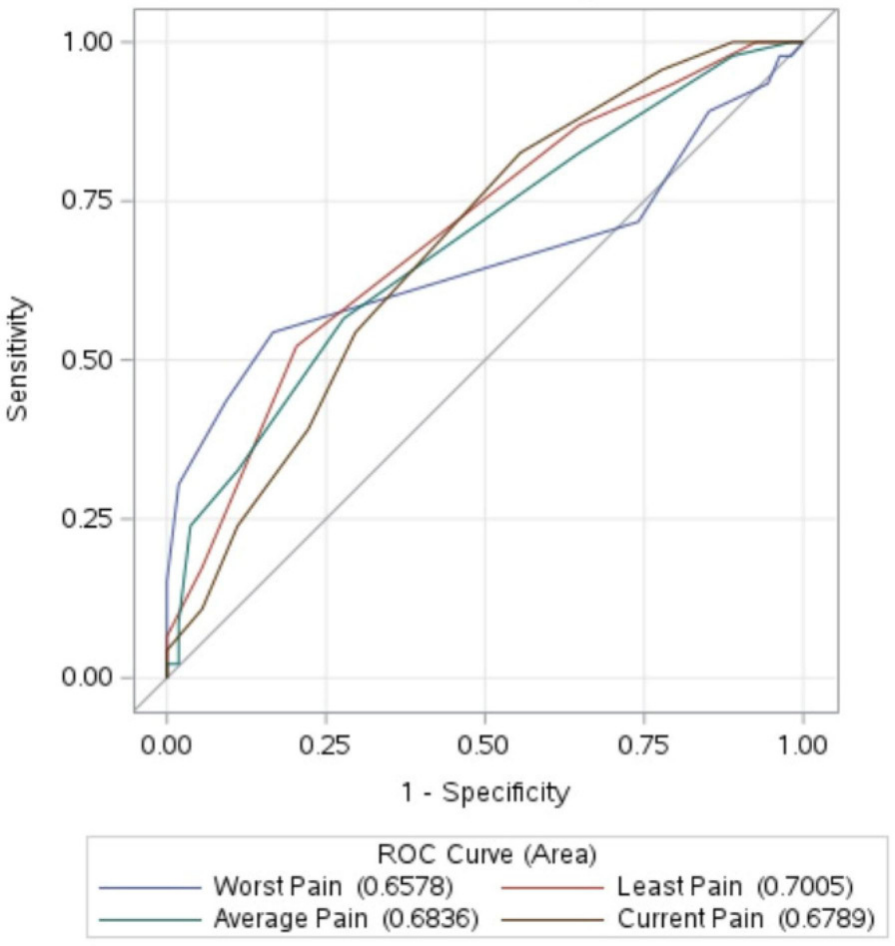
Area under the curve comparison for detecting pain improvement. ROC, Receiver Operating Characteristic, *p* > 0.05 for comparison.

AUC analysis demonstrated that pain severity measures effectively captured responsiveness to improvement, with values ranging from 0.66 to 0.70 (Figure 2). No statistically significant differences were found in AUC among the NRS measures with different references (worst, least, average, current).

## Discussion

In this study, we triangulated various approaches to estimate MIDs and assess responsiveness to change for NRS measuring menstrual pain severity. We found that the MID estimates were close to 1 point on a 0–10 scale of menstrual pain severity and the 0–10 NRS was responsive in detecting menstrual pain improvement.

We estimated the MIDs by triangulating distribution-based and anchor-based methods, leveraging their complementary strengths. As shown in Table 2, anchor-based estimates were consistently smaller than those derived from distribution-based methods. To account for potential measurement error, we set the lower bound using distribution-based estimates and selected values larger than the anchor-based estimates. Our MID estimate is similar to the SEM of NRS for menstrual pain severity reported in a Brazilian study, which was 0.97 (13). However, our MID estimates were smaller than some reported in the literature. For example, several studies on other chronic pain conditions (e.g., musculoskeletal pain, neuropathy) showed MIDs of about 2 points on a 0–10 scale (11, 12, 45). A study of patients with moderate-to-severe surgically confirmed endometriosis suggested 4-point as the clinically important difference for “worst pain in the previous 24 h” (15). Two studies outside of United States suggested that an approximately 3 point change represents a clinically important difference for menstrual pain or pelvic pain (13, 14). These differences may be due to variations in pain conditions, study sample characteristics such as initial pain severity, and methodologies, further suggesting that MID estimates can be context-dependent. MID estimates from other chronic pain populations may not apply to menstrual pain. Some previous studies had baseline pain entry requirements, resulting in higher mean baseline pain than our study population (11, 14, 15, 45). For individuals with high pain severity, a larger change may be needed to be minimally important. Methodological differences also exist between our study and previous ones. The endometriosis study grouped “very much improved,” “much improved,” and “minimally improved” into one category (15), whereas we treated these as distinct categories for MID estimation. A Brazilian study used 2.77 SEM as the estimate for the clinically important difference in menstrual pain, while we used 2 SEM as the upper limit for our MID estimates (13). Another study across several countries reported a similar 0.5 SD (i.e., approximately 1.5 points) aligning with our findings (14). However, unlike our approach, they did not use 0.5 SD as the upper bound for the MID estimate. Their estimates may be clinically meaningful, but exceed the threshold for minimal importance, indicating moderate or substantial importance. Compared to the endometriosis study where participants were followed up at 3 and 6 months (15), we followed up with participants 24 h after the initial assessment to reduce recall bias for the retrospective global rating of change. Importantly, evidence-based reports using a 0-to-10 NRS to compare pain treatments have defined small, moderate, and large differences as 0.5, 1.0, and 2.0 points (46). Notably, the majority of our MID estimates in Figure 1 fell into the 0.5–1.0 point range.

Regarding responsiveness, we found that the 0–10 NRS of pain severity was responsive in detecting pain improvement, as supported by the previous literature on other chronic pain conditions (16). As shown in Figure 2, the AUCs were within a similar range to those reported in other studies that used retrospective global ratings of change (47–49), where AUC values are typically lower than in studies evaluating diagnostic tests for disease detection. While the NRS was responsive to pain improvement, it was less so for pain worsening. In our study, pain change scores for the improvement group had moderate effect sizes measured by SRM, with magnitudes above the MID. For the worsening group, the NRS showed minimal to small effect sizes, with magnitudes below the MID. The larger SRMs for improvement compared to worsening might be due to the typical improvement of menstrual pain during a menstrual period, with or without treatment. Interestingly, interventional studies on other chronic pain conditions also suggest that self-report pain measures are more responsive to pain improvement than worsening (15, 47). Additionally, scales for symptoms other than pain—such as fatigue, depression, and anxiety—have also proven better at detecting improvement than worsening (48, 50, 51). Individuals undergoing treatment might expect improvement, making them more attuned to positive changes. When comparing NRS with different references (worst, least, average, current), we found MID estimates were largely consistent. The MID for current pain was slightly larger, likely due to the lower test-retest reliability coefficient for the current pain rating used for the SEM calculation. We did not find any statistically significant differences in responsiveness to change across different references. This finding differed from that of a study of patients with chronic pain undergoing a pain management program, where researchers showed that current pain was more responsive to detect pain improvement than least, average, and worst pain measures (52). Menstrual pain is episodic and can vary within a day and across days. Findings from other chronic pain conditions may not be applicable to menstrual pain. In addition, our sample size may limit our ability to detect any statistically important differences in responsiveness across different references.

Our study has several strengths. We used multiple approaches to estimate MID and assess responsiveness, enhancing the robustness of our findings. Additionally, we employed a 24-hour retrospective recall, minimizing recall bias and providing a more reliable assessment of pain changes over a short time frame.

We acknowledge several limitations. First, our analysis was based on data from a single study cohort. Although literature supports the legitimacy of estimating MID and responsiveness in observational studies and real-world settings (53), further research across multiple cohorts and within clinical trial contexts is needed to validate these findings. Second, in some distinct anchor categories, the sample size was small. We excluded subgroups with fewer than 10 samples from MID estimations, as estimates based on such small numbers would be unreliable (23). Third, our sample likely included participants who used a range of treatment approaches as well as those who did not use any. We did not assess specific interventions for menstrual pain due to the wide variety of both pharmacological and non-pharmacological strategies people use for menstrual pain and variability in their effectiveness across individuals (54, 55). While our findings offer real-world evidence on the NRS's performance, future research should evaluate its responsiveness and MID estimates in different settings to enhance generalizability and clinical relevance. Fourth, at baseline, participants were on days 1–3 of their menstrual cycle. Due to the limited sample size, we did not conduct stratified analyses by cycle day, which could help determine whether MID and responsiveness estimates vary by timing within the cycle. Fifth, although we triangulated distribution- and anchor-based approaches in this study, we did not incorporate qualitative data—such as asking participants what kinds of changes in menstrual pain they perceive as meaningful—which could offer valuable, person-centered insights (56). It is possible that a larger change is needed for participants to perceive it as truly meaningful and further investigation is warranted.

This study has implications for future research. Additional studies with larger and more diverse samples and in clinical trials are needed to validate our findings and improve their generalizability. Our MID estimates were derived from remotely administered surveys but may be cautiously considered for clinical contexts, with appropriate validation. Comparing 0–10 NRS with other outcome measures and scales, such as the Visual Analog Scale and the pain interference scale, would also be informative. More granular assessments of menstrual pain severity—such as multiple daily ratings using ecological momentary assessment—particularly during the days leading up to and at the onset of menstruation, may better capture pain exacerbation and enhance the evaluation of MID and responsiveness. Additionally, incorporating qualitative data from participants could help clarify what constitutes a minimally important difference from a person-centered perspective.

The findings of this study have implications for clinical practice. Identifying a MID of approximately one point on a 0–10 numerical rating scale provides clinicians with a clear, quantifiable threshold to gauge meaningful improvement in a patient's menstrual pain. This benchmark can be used to assess the efficacy of interventions and inform treatment adjustments. Given that the NRS demonstrated responsiveness to menstrual pain improvement, clinicians can use this tool to track patient progress over time, enabling timely modifications to management strategies.

## Conclusion

As the NRS takes integer values between 0 and 10, the MID for menstrual pain severity can be rounded up to 1 point for practical use. The NRS is responsive in detecting menstrual pain improvement. These findings can inform the design and interpretation of studies testing interventions for menstrual pain and guide clinicians in interpreting the magnitude of treatment effects.

## Data Availability

The raw data supporting the conclusions of this article will be made available by the authors, without undue reservation.
